# The Climatologist

**Published:** 1892-06

**Authors:** 


THE CLIMATOLOGIST. A monthly journal of medicine. Edited by John M. Keating, M. D., Frederick A. Packard, M. D., and Charles F. Gardiner, M. D. W. B. Saunders, Publisher, 913 Walnut Street, Philadelphia, Pa. Yearly subscription, $2.00. Single number, 20 cents.

This journal, as its name implies, is devoted to medicine in its relation to Climate, also to Mineral Springs, Diet, Preventive Medicine, Race, Occupation, Life Insurance, and Sanitary Science. The April number is now before us, and is well charged with interesting matter. A few of these articles may be mentioned: "Vichy," by Dr. C. E. Cormack; "The Healing of Tuberculosis," by Dr. Wm. Osler; "The Injustice of Regarding suggested means for Treating Phthisis as Attempts to Discover a Specific Cure," by Dr. H. F. Williams; "The Surgical Treatment of Acute and Chronic Empyema," by Dr. Morris H. Richardson, and "Verbum Sat. Sapienti, No. 2," by Dr. John M. Keating.

